# Prospective randomized controlled study on improving sleep quality and impact of zolpidem after total hip arthroplasty

**DOI:** 10.1186/s13018-019-1327-2

**Published:** 2019-09-03

**Authors:** Hirose Shakya, Duan Wang, Kai Zhou, Ze-Yu Luo, Suraj Dahal, Zong-Ke Zhou

**Affiliations:** Department of Orthopedics, West China Hospital, Sichuan University, 37# Wuhou Guoxue Road, Chengdu, 610041 People’s Republic of China

**Keywords:** Total hip arthroplasty, Sleep quality, Anxiety, Depression, Quality of recovery

## Abstract

**Background:**

Total hip arthroplasty (THA) is a proven surgical option for patients with end-stage osteoarthritis in terms of improved function and pain relief. A prospective study was conducted to examine and evaluate the effect and impact of zolpidem postoperatively on the sleep quality, pain alleviation, and quality of life of patients who underwent total hip arthroplasty.

**Methods:**

A total of 160 patients was randomized 1:1 to receive either zolpidem or placebo 2 days preoperative to 5 days postoperatively. Pain scores using visual analog scale (VAS), sleep quality using Pittsburgh Sleep Quality Index and Epworth Sleepiness Scale, quality of life using QoR-40, and Hip disability and Osteoarthritis Outcome Score were recorded. The total amount of opioid analgesics and antiemetics taken was recorded as well.

**Results:**

Patients in the intervention group had higher VAS score and took less analgesic and antiemetic. Moreover, the study demonstrated that QoR-40 was higher and Hip disability and Osteoarthritis Outcome Score had relatively lower mean value (*P* < 0.05) in the treatment group. Pittsburgh Sleep Quality Index and Epworth Sleepiness Scale were also lower in the treatment group (*P* < 0.05).

**Conclusion:**

Patients taking zolpidem achieved greater improvement in the quality of life and reported better satisfaction. The study demonstrated zolpidem 10 mg can improve sleep quality effectively, relieve pain, increase early range of motion and muscle strength, reduce the perioperative anxiety and depression, and improve perioperative experience and satisfaction, thereby reducing the hospital stay and medical costs and promote the rapid recovery and quality of life.

**Trial registration:**

The trial was registered on Chinese Clinical Trial Registry, ChiCTR-IOR-16007861.

## Background

Patients with osteoarthritis commonly complain of sleep disturbance that may be due to pain and may also contribute to pain. Osteoarthritic (OA) hip pain is commonly alleviated by total hip arthroplasty (THA) [1]. The goals of total hip arthroplasty (THA) include relieving pain and improving physical function [2, 3].

A successful THA depends on multiple factors, such as patient and prosthesis selection, surgical technique, pain management, and functional exercise [4]. Generally, we can evaluate, qualitatively and quantitatively, how much patients benefit from THA by using Epworth Sleepiness Score (ESS), Pittsburgh Sleep Quality Index (PSQI), Hip disability and Osteoarthritis Outcome Score (HOOS), and visual analog scale (VAS) questionnaire to cover pain relief, functional recovery, and improvement in quality of life.

Zolpidem is short for zolpidem tartrate tablets (STILNOX, Sanofi-Synthelabo, Ltd, France) is a new generation of non-benzodiazepine drugs, which can effectively improve the quality of postoperative sleep in patients undergoing hip and knee arthroplasty and improve the proportion of postoperative fast eye movement and slow-wave sleep but do not change the sleep structure [5, 6]. Zolpidem is a proven safe and effective drug and is used for transient occasional insomnia and chronic insomnia and for repairing disrupted sleep [7]. It helps patients fall asleep quickly and wake up without movement disorders [4].

## Methods

The prospective randomized, double-blind, controlled, and single-center study is a branch project of the Public Welfare Industry Research Project “Evaluation of the Safety and Effectiveness of Joint Replacement” (No. 201302007) hosted by the Department of Joint Surgery. At the same time, the China Clinical Trial Registration Center carries out prospective registration (registration number ChiCTR-IOR-16007861). The study was conducted in patients waiting for first-line primary unilateral total hip arthroplasty (THA) in the Joint Surgery Department, West China Hospital, Sichuan, between September 2015 and December 2016. The study was approved by the Medical Ethics Committee of West China Hospital of Sichuan University. Prior to the study, the informed consent from patients and/or their relatives were taken from those who were willing to participate.

The study was conducted by a team consisting of a professor, an attending doctor, and a postgraduate student. All staffs in this study were blinded to the study.

A total of 179 participants were eligible; eight patients withdrew as they were no longer interested in the study. Five patients had incomplete perioperative functional activity and pain score data. Three patients were reluctant to take sleep after surgery, and three patients had postoperative poor quality of sleep so they were excluded for the trial. Consequently, 160 participants aged between 26 and 83 years (mean, 66.06 years; standard deviation (SD), ± 8.44) were included, of whom 82% were women and 12% were men. The participants were then randomly allocated into two groups using the Random Number Generator which randomly assigned information into a sealed opaque envelope; the subjects were then randomly allocated into two groups using the Random Number Generator which randomly assigned information into a sealed opaque envelope. Three groups were involved in the current study: researchers, investigators, and outcome evaluators. The study was designed in such a way that each group was blinded to the other group’s assignment. The researchers did not participate in the distribution of the sub-group information into patients’ research kit. Investigators who did not participate in the outcome evaluation issue medication orders (medications or non-medications) and document medication adherence. Drugs were loaded into the opaque no description package. Outcome evaluators did not know random information and grouping information, mainly by senior physicians involved in clinical research to complete. The two groups are termed blank control group and treatment group. The treatment group received either zolpidem or placebo (control group) preoperatively 2–5 days postoperatively.

### Intervention

All patients were given perioperative sleep care education according to “Chinese Adult Insomnia Diagnosis and Treatment Guide” and provided available amenities. Other factors implemented were reducing personnel visits and night treatment and keeping the ward quiet.

Patients in different groups were treated with different interventions. The control group was treated only according to the general procedure of rapid perioperative rehabilitation of THA, and no relevant sleep medication was applied. In the treatment group, besides conventional rapid rehabilitation by a dedicated researcher, a doctor’s advice was issued zolpidem 10 mg for 2 days preoperatively to 5 days after surgery, 30 min prior to going to bed.

### Surgical methods

All operations were carried by the same medical team. All anesthesia was induced by intravenous, injection. Sufentanil, propofol, and atracurium combined with midazolam were used to induce general anesthesia.

Patients with postoperative pain were given either oral diclofenac sodium enteric-coated tablets 50 mg q12h, or celecoxib capsule 200 mg bid, ketorolac tromethamine10 mg q12h, pethidine hydrochloride 40 mg, or 40 mg intramuscular analgesia (parecoxib sodium for injection) given to patients with severe postoperative pain for whose VAS pain exceeded 7.

### Baseline information

The baseline information of the patients included basic information such as sex, age, height, weight, and BMI which is detailed in Table 1.

**Table 1:** Baseline information of the patients

Variable	Control group	Treatment group
Age	67.66 ± 9.14	64.47 ± 7.39
Sex		
Male	20	9
Female	60	71
Height(m)	1.56 ± 0.08	1.55 ± 0.06
Weight (kg)	60.75 ± 12.77	64.48 ± 9.95
BMI (kg/m^2^)	24.99 ± 4.20	26.59 ± 3.51

*BMI* body mass index

### Quality of sleep

Sleep quality can be measured by using both subjective and objective methods. The former mainly includes Epworth Sleepiness Score (ESS) and Pittsburgh Sleep Quality Index (PSQI).

ESS which is a survey generally measures daytime sleepiness of one person and is a self-administered and validated questionnaire with different points allocated to assess the state of excessive daytime sleepiness (ESS > 6 points for drowsiness, > 11 points for excessive drowsiness, > 16 prompts dangerous) is used. ESS is collected preoperatively and 1 day, 3 days and 5 days after the operation.

The Pittsburgh Sleep Quality Index (PSQI score) a self-rated questionnaire that assesses sleep quality and disturbances over a 1-month time interval comprised of 19 self-assessments and categorized into seven dimensions (quality of sleep, fall asleep, sleeping time, sleep efficiency, sleep disorders, hypnotic medications, and daytime dysfunction) with 0–3 points for each dimension and the accumulated scores for all dimensions are the total score of the PSQI scale. The total score ranged from 0 to 21, with PSQI > 7 as the quality of sleep, which was inversely proportional to the quality of sleep. Data were collected preoperatively and postoperatively after 3 weeks and 3 months.

### Pain protocol

Visual analog scale (VAS) pain score was selected as the primary efficacy parameter with points ranging from 0 to 10. The five levels of pain assessment with different points allocated were the “very painful” 8–10 points, “painful” 6–8 points, “not so painful” 4–6 points, “little pain” 2–4 points, “no pain” assignment 0–2 points. VAS pain scores at rest, night, and ambulation were evaluated before the surgery and at postoperative days (POD) 1, 3, and 5. Night pain was assessed on the following day. In particular, rest, ambulation, and nocturnal VAS pain scores which were attained respectively provide more useful and detailed information about patients’ pain sensations after surgery. This helped us to judge or estimate the severity of pain at rest, on movement, and at night, respectively [8].

### Quality of life

The Hip disability and Osteoarthritis Outcome Score (HOOS) questionnaire were intended to assess the patient’s opinion about their hip and associated problems and also evaluation of symptoms and functional limitations related to the hip during a therapeutic process. Data was collected preoperatively, 3 week and 3 months, respectively.

Quality of recovery-40 (QoR-40) evaluated the recovery of patients’ physical and psychological symptoms, during short-term perioperative period and recovery of short-term follow-up. Satisfaction and anxiety and depression satisfaction were recorded before and after surgery for 3 weeks and 3 months, respectively. Five grades were used for assignment: very satisfied were given 8–10 points, satisfied with 6–8 points, basically satisfied with 4–6 points, not quite satisfied with 2–4 points, not satisfied with 0–1 points, personnel in the postoperative 1 day, 3 days, 5 days to collect data.

### Anxiety and depression

The Hamilton Anxiety and Depression Scale was used to assess the severity of anxiety and depression in patients (may be severe anxiety ≥ 29 points, definite anxiety ≥ 21 points, there must be anxiety ≥ 14 points, there may be anxiety > 7 points, no anxiety symptoms < 7 points). Data was collected from preoperative to postoperative 1 day, 3 days, and 5 days.

### Statistical method

Data Processing Statistical analysis using SPSS19.0 software for data analysis was used. Data were expressed as mean ± standard deviation (SD) or *n*%, chi-square test was used to compare the incidence of other indicators such as incidence of hospitalization, and one-way analysis of variance (ANOVA) was used to measure the length of hospital stay. Multivariate correlation analysis using logistic regression analysis, *P* < 0.05, considered the difference was statistically significant.

## Results

From September 2015 to December 2016, 160 patients completed the data collection of research-related indicators. The baseline information of the patients in the trial which are shown in Table 1 indicated there was no significant difference in age, body weight, height, and BMI in the control group and treatment group (zolpidem 10 mg) whereas statistically significant difference was noted in hospitalization time in the two groups. The treatment group patients were hospitalized for the shortest time and had the fastest time to go to ground for the first time and walk independently for the shortest time (Table 2).

**Table 2:** Hospitalization time (days)

Control group	Treatment group	*P* value
7.24 ± 3.23	5.44 ± 1.76	< 0.05

**Fig. 1:**
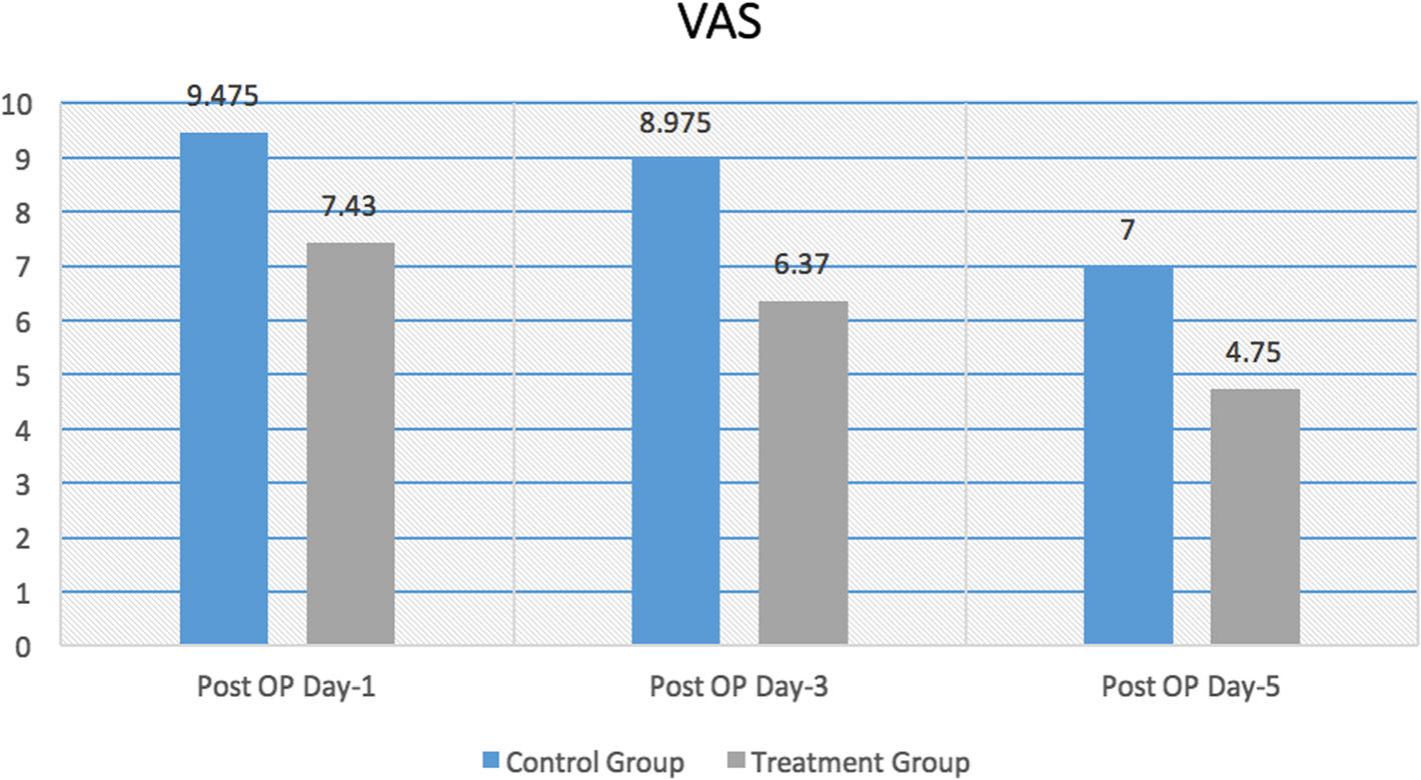
Visual analog scale for pain (VAS)

The statistically significant difference was noted in VAS between two groups (Fig. 1) with patients in the treatment group had a relatively lower score as compared to the control group during postoperative days 1, 3, and 5.

**Fig. 2:**
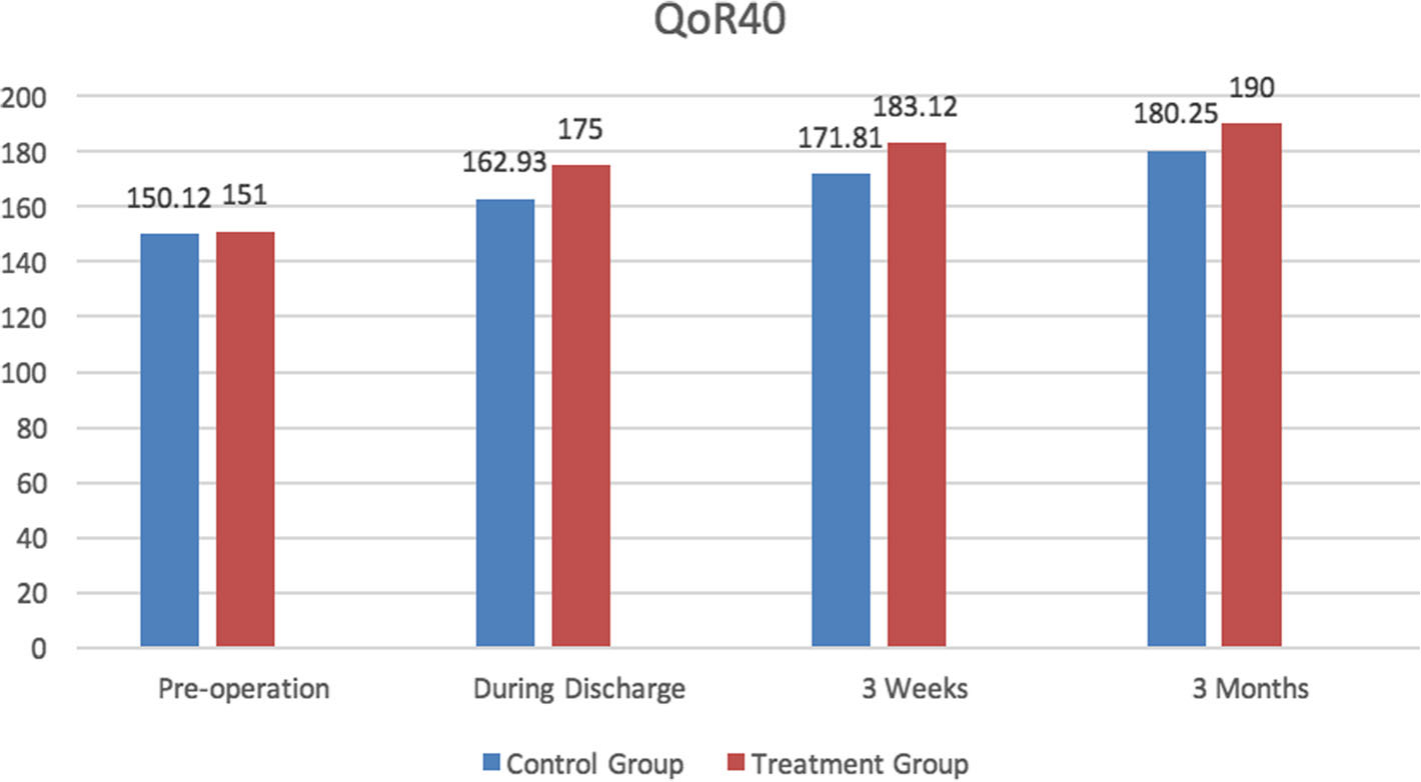
Quality of recovery score (QoR40)

The preoperative QoR-40 score in two groups was not statistically significant during pre-operation while the scores were statistically significant during discharge, 3 weeks and 3 months after surgery. The QoR-40 scores of patients in the treatment group were higher than the control group (Fig. 2).

Table 3 summarizes a significant difference in the five dimensions of HOOS in the control group and treatment group pre- and postoperatively. The treatment group showed relatively lower mean value with statistically significant result (*P* value is < 0.05) compared to the control group.

**Table 3:** Hip disability and Osteoarthritis Outcome Score (HOOS)

	Control group	Treatment group	*P* value
Symptoms			
Pre-operation	19.31 ± 3.42	19.06 ± 3.23	0.635
Post-operation			
3 weeks	14.68 ± 3.49	10.36 ± 3.23	< 0.001
3 months	10.31 ± 3.33	8.36 ± 3.23	< 0.001
Pain			
Pre-operation	26.43 ± 5.32	26.06 ± 5.07	0.649
Post-operation			
3 weeks	20.00 ± 5.37	16.06 ± 5.67	< 0.001
3 months	13.62 ± 5.53	11.06 ± 5.07	0.0027
Activities of daily living			
Pre-operation	44.68 ± 9.31	44.43 ± 9.23	0.8648
Post-operation			
3 weeks	34.81 ± 8.58	25.43 ± 9.23	< 0.001
3 months	22.56 ± 7.28	16.43 ± 9.23	< 0.001
Sport/Recreation			
Pre-operation	21.00 ± 2.38	20.75 ± 2.29	0.5002
Post-operation			
3 weeks	17.50 ± 2.41	14.75 ± 2.29	< 0.001
3 months	13.93 ± 2.31	11.75 ± 2.29	< 0.001
Quality of life			
Pre-operation	12.750 ± 1.307	12.43 ± 1.28	0.1288
Post-operation			
3 weeks	10.31 ± 1.26	8.03 ± 1.28	< 0.001
3 months	7.81 ± 1.43	6.83 ± 1.28	< 0.001

PSQI between two groups during the preoperative period in seven dimensions showed no significant difference while the scores were lower postoperatively in the treatment group. The statistically significant result was observed in all dimensions 3 weeks after surgery. Similarly, sleep quality, sleep latency, and sleep duration also showed significant results for 6 weeks postoperatively (Table 4).

**Table 4:** Pittsburgh Sleep Quality Index (PSQI)

	Control group	Treatment group	*P* value
Sleep quality			
Pre-operation	1.25 ± 0.43	1.18 ± 0.39	0.3421
Post-operation			
3 weeks	1.62 ± 0.48	0.68 ± 0.54	< 0.001
3 months	1.06 ± 0.43	0.53 ± 0.55	< 0.001
Sleep latency			
Pre-operation	1.93 ± 0.75	1.87 ± 0.70	0.5872
Post-operation			
3 weeks	2.06 ± 0.55	1.33 ± 0.55	< 0.001
3 months	1.68 ± 0.46	1.03 ± 0.63	< 0.001
Sleep duration			
Pre-operation	1.68 ± 0.46	1.62 ± 0.48	0.4085
Post-operation			
3 weeks	2.18 ± 0.39	1.56 ± 0.49	< 0.001
3 months	1.62 ± 0.48	1.12 ± 0.33	< 0.001
Sleep efficiency			
Pre-operation	1.12 ± 0.33	0.75 ± 0.43	< 0.001
Post-operation			
3 weeks	1.81 ± 0.63	1.06 ± 0.24	< 0.001
3 months	0.75 ± 0.56	0.68 ± 0.46	0.4454
Sleep disturbance			
Pre-operation	1.25 ± 0.43	1.18 ± 0.39	0.3421
Post-operation			
3 weeks	1.81 ± 0.63	1.43 ± 0.61	< 0.001
3 months	0.87 ± 0.60	0.68 ± 0.58	0.0480
Hypnotic medicine			
Pre-operation	0.25 ± 0.43	0.18 ± 0.39	0.3421
Post-operation			
3 weeks	1.31 ± 0.58	0.62 ± 0.700	< 0.001
3 months	0.31 ± 0.58	0.18 ± 0.39	0.1153
Daytime dysfunction			
Pre-operation	0.93 ± 0.55	0.93 ± 0.66	1.000
Post-operation			
3 weeks	1.68 ± 0.58	0.81 ± 0.63	< 0.001
3 months	0.81 ± 0.53	0.56 ± 0.49	0.0025

EES scale of the two groups was statistically significant at days 1, 3, and 5 postoperatively. The indices of EES in the treatment group were significantly lower compared to the control group. The patients in the treatment group had the lowest sleepiness index (EES), which was significantly different from those in the control group (*P* < 0.05).

**Fig. 3:**
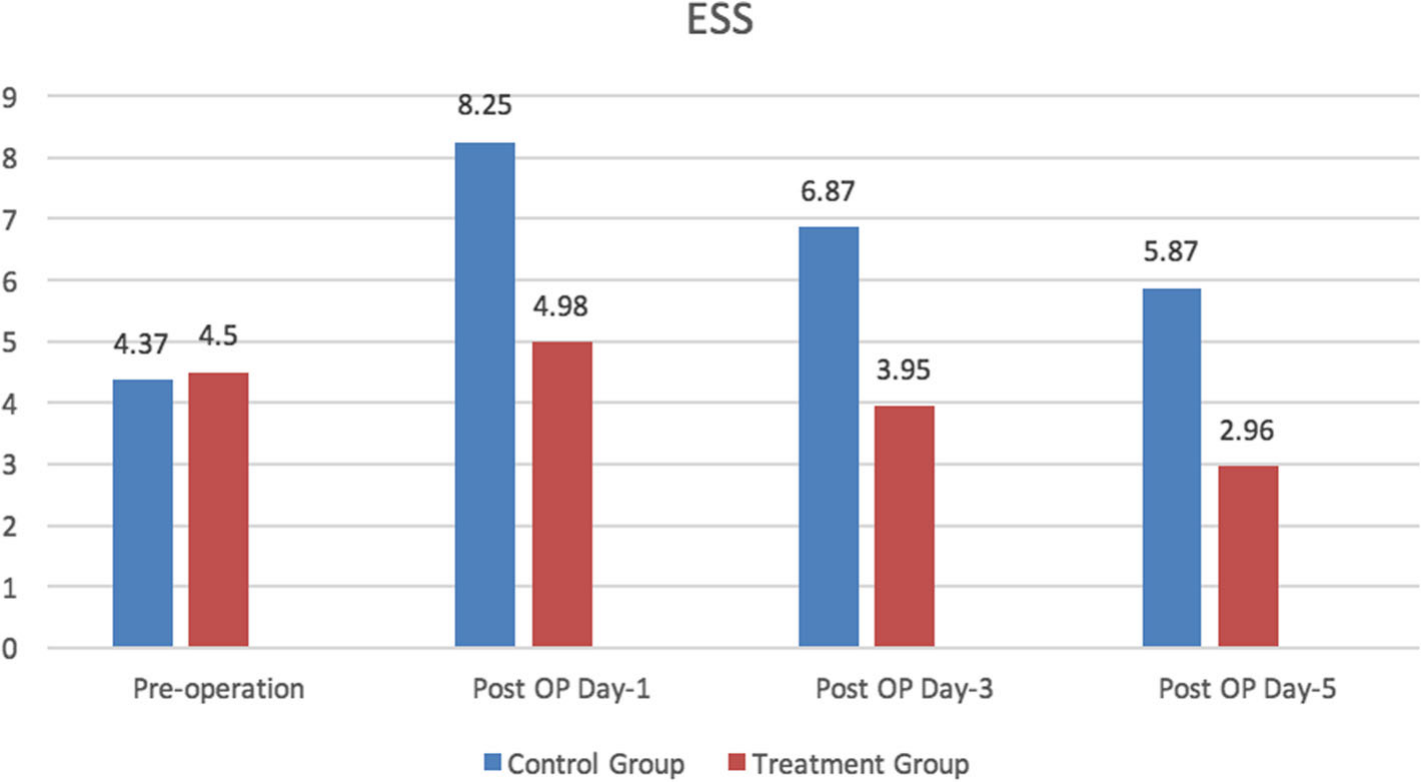
Epworth Sleepiness Scale (ESS)

**Fig. 4:**
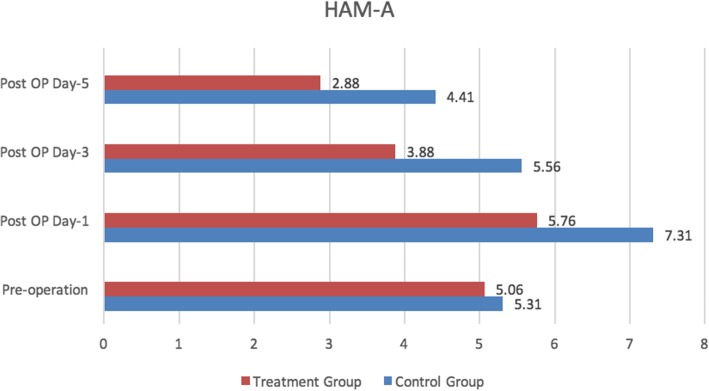
Hamilton Anxiety Rating Scale (HAM-A)

**Fig. 5:**
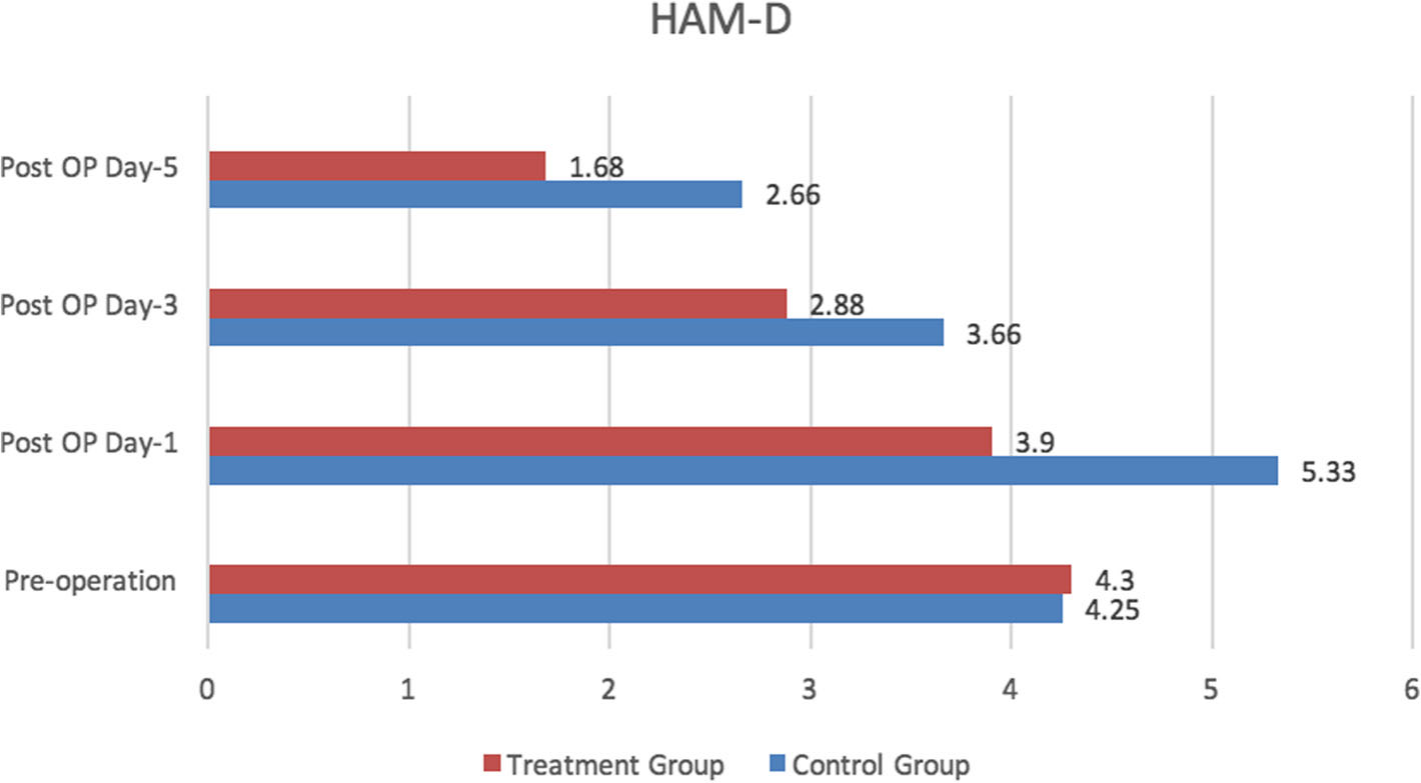
Hamilton Rate Scale for Depression (HAM-D)

No significant difference was seen in the Hamilton Anxiety Rating Scale (HAM-A) and Hamilton Rate Scale for Depression (HAM-D) scores between the two groups prior to the operation (Fig. 3). The HAM-A and HAM-D scores were significantly lower in the treatment group in days 1, 3, and 5 after the operation as compared to those in the blank control group, indicating the result statistically significant (Figs. 4 and 5).

## Discussion

Modern total hip arthroplasty (THA) has tremendously ameliorated the prognosis for patients with end-stage hip disease [9], and it is the most effective surgical modality for the treatment of hip degenerative or rheumatoid arthritis disease that is refractory to conservative treatment to minimize hip pain and improve patient quality of life [10]. According to statistics, the USA has annually more than one million joint replacements, with the accelerated aging and the growth of its demand day by day. With the gradual maturity and wide application of total hip arthroplasty, patients and doctors are increasingly demanding perioperative management and experience. Therefore, efficient and planned perioperative multi-modal management and perioperative management for rapid recovery are necessary. However, the study reported that the majority of patients undergoing major orthopedic surgery experienced severe deterioration of their sleep quality, seriously affecting perioperative functional exercise and rapid recovery and reducing perioperative [11, 12]. Researchers have speculated that sleep disturbance in older populations may, in part, be secondary to chronic medical conditions such as osteoarthritis (OA) [13, 14], and pain directly leads to sleep disruption or even deprivation and, in turn, poor-quality sleep aggravates pain sensation. There seems to be a vicious circle: pain—poor-quality sleep, intensified pain—even poorer-quality sleep [4].

Studies have shown that nocturnal pain directly causes sleep disorders, even sleep deprivation, which in turn can lead to pain sensitivity, low-quality sleep, worse pain, and worse cycle of lower quality sleep, which can greatly affect early rehabilitation of patients [15].

Other studies have shown postoperative pain is the most common cause of sleep quality in patients at night, often causing nighttime wakefulness, nightmares, or even unable to sleep [16].

Low-quality sleep can affect subjective experience and satisfaction of patients, prolong hospitalization, and increase the financial burden on the patients’ families, which runs counter to the concept of accelerated rehabilitation of the hip and knee [12, 17]. Still further, persistent postoperative low-quality sleep can lead to reduced pain thresholds and increased nighttime pain, which in turn can further affect nighttime sleep quality.

At the same time, postoperative sleep disorders in patients with total hip arthroplasty reduce postoperative pain tolerance and increased sense of pain experience, thereby increasing postoperative pain in patients.

Cremeans-Smith et al. prospectively enrolled 110 patients undergoing total hip and knee arthroplasty to assess the relationship between postoperative sleep disorders and pain and found that improving perioperative sleep quality can reduce postoperative pain and increase patient satisfaction and recovery [12]. Likewise, Sleep time, while improving the quality of sleep, can reduce hospital stays, speed early recovery, and improve patient satisfaction [18].

At present, the research on the relationship between perioperative rapid rehabilitation and quality of sleep is relatively lacking. Numerous studies have monitored the sleep of patients awaiting THA or have measured whether sleep disturbance diminishes after surgery. This study found significant improvements in this sleep measure at all post-surgery measurement points. Therefore, it led to believe that sleep quality is one of the important factors influencing THA’s results. Improved sleep quality at the early stage after THA may provide additional pain relief with the current analgesic protocol. This result is consistent with the previous study carried by Gong et al. [4].

At the present stage, the evidence of the lower level of evidence-based medicine cannot guide the improvement of clinical sleep and achieve the goal of rapid rehabilitation.

Therefore, multimodal sleep management with non-benzodiazepines in combination with psychological interventions can improve perioperative sleep quality and promote accelerated recovery in patients undergoing total hip arthroplasty.

Miller et al., 50 cases of patients after total hip or knee arthroplasty, with body motion recorder to detect perioperative sleep quality and analysis of postoperative quality of sleep and pain, found that effective postoperative analgesia can improve postoperative sleep quality and increase overall sleep time, while improving quality of sleep can reduce hospital stays, speed early recovery, and improve patient satisfaction [18].

After investigating postoperative orthopedic surgery pain in six medical centers in four countries, the report showed 81% of orthopedic patients undergoing severe night pain [19].

Robert and others prospectively enrolled 68 patients undergoing arthroscopic surgery to study the relationship between sleep quality and pain, fatigue index, and analgesic drug use, and found that postoperative quality of sleep and fatigue index is an important factor affecting postoperative pain and early rehabilitation of patients. Poor quality of sleep increases daytime fatigue in patients [4].

The present study found that patients’ nocturnal quality of sleep was positively correlated with daytime sleepiness index and fatigue index and that zolpidem 10 mg reduced the degree of daytime fatigue and somnolence by improving sleep quality indicating zolpidem is an effective drug in improving postoperative sleep quality in patients undergoing total hip arthroplasty. Valente et al. found that proper and short-term application of zolpidem has been proved to be safe and non-addictive [7].

Zolpidem quickly induces the patient to quickly fall asleep. In the current study, patients are asked to take medication half an hour before falling asleep. They were also asked to take the inevitable activities under the supervision of nurses after administering the medication to avoid the risk of falls. The current study showed the intervention of zolpidem had significantly reduced the hospital stay to 6 days which lowered the financial burden of a patient. The previous findings by Zhang et al. showed the mean hospital stay for patients who underwent unilateral THA was 17 days [20].

At the same time, through the PSQI of the patients after 3 weeks and 3 months of sleep quality, zolpidem 10 mg group patients with the fastest recovery of sleep quality. Similarly, the present study carried by Gong et al found that zolpidem can significantly improve postoperative pain, sleep quality, and joint activity and confirmed a positive correlation between activity and sleep quality [4]. Other studies showed zolpidem improves nighttime sleep quality and reduces daytime fatigue index [5]. The study showed medication with zolpidem postoperatively had a promising result and future studies should be directed at the long-term efficacy and safety of zolpidem for patients undergoing THA.

The study also confirmed that patients with postoperative sleep quality and joint mobility were positively correlated.

Studies have shown that anxiety and pain are independent risk factors for postoperative sleep quality, and conversely, low-quality sleep increases the postoperative anxiety and depression in patients [21, 22]. Unfamiliar environment and ward accommodation, postoperative rehabilitation exercises and rehabilitation anxiety, and a series of other factors lead to preoperative and postoperative anxiety. This study found that 10 mg zolpidem significantly improved anxiety and depression during hospitalization.

## Conclusion

Sleep quality is impaired both before and after THA surgery to varying degrees. Relieving pain from osteoarthritis as a result of THA improves sleep quality which in turn contribute to the improved quality of life and day-to-day functioning seen after THA.

Short-term application of zolpidem can effectively improve the quality of perioperative sleep in patients undergoing THA under rapid rehabilitation management. It reduces perioperative nighttime, walking, rest, and pain, effective in alleviating pain resulting less doses of opioids during the perioperative period.

It is also effective in improving perioperative muscle strength and hip joint activity resulting in increased perioperative satisfaction and recovery confidence promoting patients to exercise in the morning and walk independently which in turn contribute in rapid recovery.

Zolpidem can relieve pain and increase early range of motion and muscle strength. It reduces perioperative anxiety and depressions and improves perioperative experience and satisfaction, thereby significantly reducing the hospital stay and medical costs and promotes rapid recovery and quality of life.

## Data Availability

Data used and analyzed in this study are available from the corresponding author on reasonable request.

## Abbreviations

BMI: Body mass index
ESS: Epworth Sleepiness Scale
HAM-A: Hamilton Anxiety Rating Scale
HAM-D: Hamilton Rate Scale for Depression
HOOS: Hip disability and Osteoarthritis Outcome Score
OA: Osteoarthritis
POD: Postoperative day
PSQI: Pittsburgh Sleep Quality Index
QoR-40: Quality of recovery-40
THA: Total hip arthroplasty
VAS: Visual analog scale
